# Integrating a Hand Held computer and Stethoscope into a Fetal Monitor

**DOI:** 10.3885/meo.2009.T0000135

**Published:** 2009-02-07

**Authors:** Mitra Ahmad Soltani

**Affiliations:** Vice-Chancellorship, Department of OB-GYN, Guilan University of Medical Sciences- Research, Iran

**Keywords:** Pocket PC, PDA, sonic aid, digital stethoscope

## Abstract

This article presents procedures for modifying a hand held computer or personal digital assistant (PDA) into a versatile device functioning as an electronic stethoscope for fetal monitoring. Along with functioning as an electronic stethoscope, a PDA can provide a useful information source for a medical trainee. Feedback from medical students, residents and interns suggests the device is well accepted by medical trainees.

Doppler devices are designed with patients’ safety in mind. Obstetrics probes emitting 2-3 MHz signals are used to detect fetal heartbeats. Each Doppler device is designed to eliminate the potential hazards to “As Low As Reasonably Achievable” (ALARA) and determined to be safe by wide margins. Even the vascular probes that emit signals in the 4-8 MHz are not safe for fetal use.^1^

Unlike Doppler devices, stethoscopes and midwifery pinards are traditional tools for collecting heart sounds on a mechanical basis; that is, they do not emit any energy into the body. Electronic stethoscopes can safely be applied on a pregnant uterus to hear and record fetal heart sounds. Brinks et al. in a table of existing market electronic stethoscopes mention the Littmann® Model 4100WS as the most versatile of these devices, with the price of $510.88 (USD). This device is capable of a 75% ambient noise reduction and includes software that provides a visual representation of the heartbeat as well as infrared transmission to another stethoscope or personal computer.^2^

Wireless mobile technology is also becoming increasingly popular in the medical community as an educational tool. Ganger and Jackson^3^ in their study of wireless handheld computers in the preclinical undergraduate curriculum found that the most useful feature of the wireless handhelds, considering accessing patient tracking applications, drug databases, and diagnostic algorithms, was preparation for the computer-based USMLE.

## Creating an Electronic Stethoscope

A PDA and a stethoscope or pinard can be used to create a device similar to an electronic stethoscope in settings where cost or other impediments make obtaining an electronic stethoscope impractical. Obtaining and modifying a PDA is affordable for middle-class medical students in Iran and has many uses. For example, it is useful in preparation for obstetrics and gynecology promotion and board exams. PowerPoint slides and other reference material, readable on a PDA, are recommended by the Iranian Ministry of Health Council for Graduate Medical Education and available for download from the Medical Education Online Resource section.^4^ A PDA can be used to calculate gestational age as well as body mass index and can provide a tool to collect and record data about patients.

**Figure 1:**
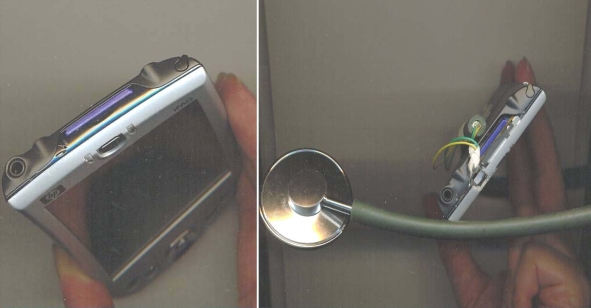
Attaching a stethoscope to the microphone of a PDA

**Figure 2:**
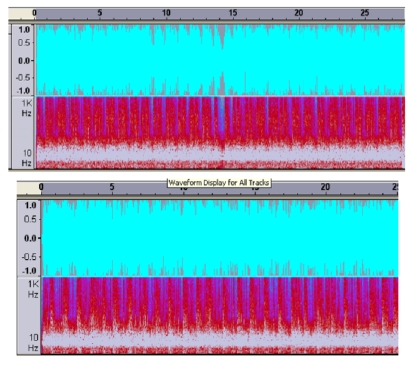
Visual comparison of digitized heart sounds from a manufactured electronic stethoscope (top) and a modified PDA (bottom).

A PDA can also be used as a real-time sound analyzer that can create and save images of heart sounds.^5^ A traditional stethoscope or midwifery pinard can be attached to the PDA microphone that captures fetal heart sounds and reduces ambient noise. An example of how these devices can be attached to the PDA is shown in Figure 1. The diagram in Figure 2 compares digitized heart sounds collected via a modified PDA with those collected by a manufactured electronic stethoscope. Once filtered by phonocardiography software,^6^ the PDA device can provide similar results to the more expensive and less versatile manufactured electronic stethoscope.

**Figure 3:**
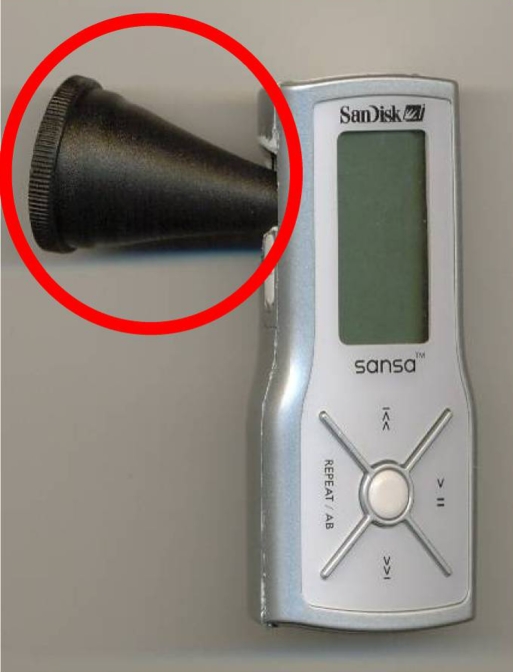
A pinard attached to an MP3 player

Pinards and stethoscopes can be attached to other devices for sound capture as well. Figure 3 demonstrates using a pinard attached to an MP3 player. The pinard acts like a bell of a stethoscope. The aptitude is less but this can be an advantage because maternal blood flow interferes with fetal heart sounds.

A manufactured electronic stethoscope and stethoscope attached mechanically to a PDA have similar features of sound intensity, but a stethoscope attached to a PDA needs software to help eliminate background noise. Pocket RTA is a relatively inexpensive (about $30 USD); this spectrum analysis software package operates on Windows Mobile PDA and can perform this task and provide tracings of fetal heart sounds.

## Evaluation

The device was introduced to a group of 28 medical trainees who were asked to assess the device's usefulness. Subjects (residents, interns and students) attended a session demonstrating the device and the files. They were asked to complete a short feedback form assessing the device.

Eighteen (64%) of the trainees indicated they would purchase and use the device while 4 (14%) would not. Six (21.4%) stated they needed more time for a decision.

The choice was correlated† with the trainee's feeling the device can be updated (r = 0.46), that it is an amusing mode of learning (r = 0.55), and it saves time (r = 0.67).† The “price” and not being a “traditional means of learning” were not related to the trainee's desire to purchase the device.

## Conclusion

This article discusses a way of modifying a PDA into an electronic stethoscope that is less expensive than a manufactured electronic stethoscope such as the Littmann® Model 4100WS. An important advantage of this method of monitoring fetal heart sounds is that it poses no danger to the fetus, as it does not use transmitted energy, like the Doppler ultrasound. Further, it offers advantage over the purely mechanical stethoscope by integrating interpretive software that can be run directly on the PDA. Comparisons of the spectrographic presentations of sounds collected by a manufactured stethoscope and the modified PDA show similar results. A pinard can be used like the bell aspect of a stethoscope and is easily attached to a digital recording device. While less sensitive than a diaphragm, it is more useful for differentiating the sharp fetal heart sounds from the maternal vascular murmurs.

Based on a small sample of trainees with different levels of experience, the device appears to be well accepted and was felt to be useful.

## Footnotes

†Pearson correlation coefficients (p < 0.01)

## References

[1] (c2007). Summit LifeDop User Manual [Internet]. [place unknown]: Fetal Doppler Facts.

[2] Brinks N, Gabler A, Moes B, Van Geest D Project Proposal and Feasibility Study: Team 6. Rhythm Reloaded [ENGR 339/340 Senior Design Engineering Project].

[3] Ganger AC, Jackson M (2003). Wireless handheld computers in the preclinical undergraduate curriculum. Med Educ Online [serial online].

[4] Soltani MA Medical decision making for common disease presentations [Internet]. Med Educ Online [serial online].

[5] Audacity Development Team (c1999-2009). Audacity: a free, cross-platform digital audio editor (Version 1.2.6).

[6] Pocket RTA User Manual [Internet].

[7] Murphy RLH, Murphy MA, Brockington G (c2002). Introduction to heart sounds: basic heart sounds [Internet].

